# Common Autoantibody among Takayasu Arteritis and Ulcerative Colitis: A Possible Pathophysiology That Includes Gut-Vessel Connection in Vascular Inflammation

**DOI:** 10.31662/jmaj.2023-0038

**Published:** 2023-05-29

**Authors:** Tsuyoshi Shirai

**Affiliations:** 1 Department of Rheumatology, Tohoku University Hospital, Sendai, Japan

**Keywords:** autoantibody, B-cell, endothelial protein C receptor, Takayasu arteritis, ulcerative colitis

## Abstract

Takayasu arteritis (TAK) is a type of large-vessel vasculitis that predominantly affects young females. The precise pathomechanism of TAK is still under investigation. In TAK, the vasa vasorum is considered to be the initial inflammatory site. Disruption of the vasa vasorum induces the entry of inflammatory cells into the vascular wall of large vessels between the media and adventitia, and infiltrated cells damage the vascular components, eventually leading to stenosis or dilatation of the affected arteries. In addition, T cells are considered key players in TAK, and myeloid cells function as effector cells. Although the roles of B cells in TAK are poorly understood, recent evidence supports their contribution to the pathogenicity of TAK. Particularly, two autoantibodies have been identified in TAK through investigation of anti-endothelial cell antibodies, and they could be involved in the maintenance of vascular inflammation. Furthermore, one of the autoantibodies, anti-endothelial protein C receptor, was shown to be present in ulcerative colitis (UC), which is genetically and clinically associated with TAK. Similar autoantibodies in inflammatory diseases with different target organs indicate a common underlying pathophysiology of these diseases, which could be characterized by the aberrant activation of B cells. This review discusses recent understanding of the pathomechanisms of TAK and UC, with a focus on the involvement of B cells and autoantibodies. The close association of UC with TAK further suggests a common etiology, and the importance of the intestinal microbiota, including dysbiosis, is also becoming known in TAK. Investigation of such common factors among TAK and UC would improve understanding of the interplay between gut and vascular inflammation, which is a new concept for developing vascular inflammation through the gut-vessel connection.

## 1. Introduction

Takayasu arteritis (TAK) is a type of large-vessel vasculitis (LVV) affecting the aorta and its major branches, which can lead to stenosis, occlusion, and dilatation of the affected lesion ^(1)^. TAK predominantly affects young females and is sometimes complicated by other autoimmune conditions ^(2), (3), (4)^. Ulcerative colitis (UC) is one of the most frequent inflammatory diseases complicated by TAK; approximately 6.4% of patients with TAK have been found to have UC ^(5)^. UC is a chronic inflammatory condition of the large intestine that is frequently associated with rectal inflammation but often extends proximally to involve additional areas of the colon ^(6)^. The onset of UC is predominant between 15 and 30 years of age, without apparent gender differences. Although epidemiological differences exist in the incidence of TAK and UC, they share common characteristics. Recently, a novel autoantibody (Ab) against the endothelial protein C receptor (EPCR) was identified in TAK ^(7)^. Anti-EPCR Abs are frequently detected in patients with TAK complicated by UC. Furthermore, anti-EPCR Abs were positive in approximately 70% of primary UC. Therefore, TAK and UC share the same anti-EPCR Abs, indicating a common pathophysiology between these diseases. This review focuses on the recent understanding of Abs and B cells in TAK and UC and discusses the possible role of gut-vessel connections in the development of LVV.

## 2. Pathophysiology of TAK

**Figure 1.**
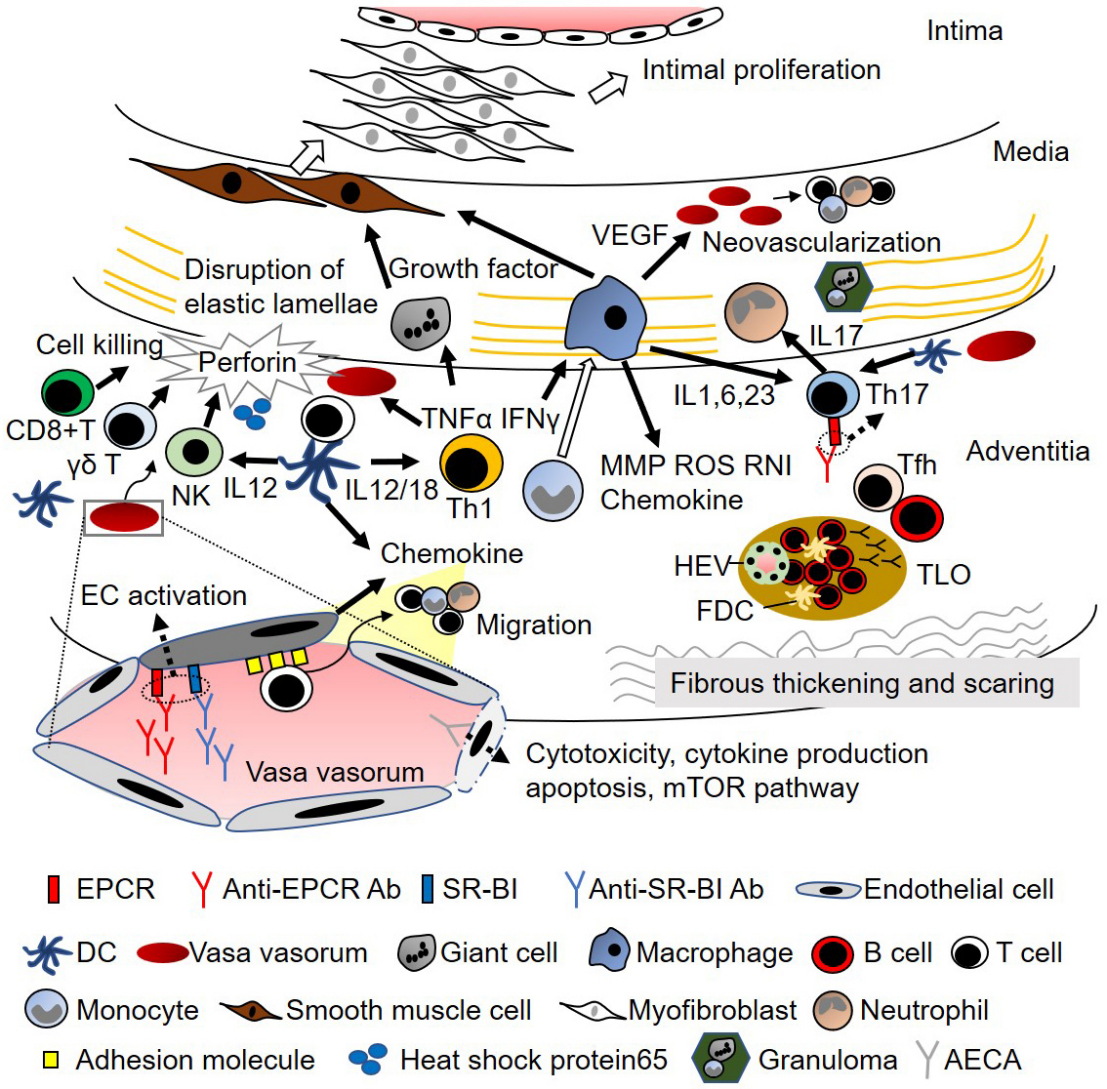
Suggested pathophysiology in Takayasu arteritis Inflammation begins around the vasa vasorum, and the involvement of heat-shock protein 65 and MHC Class I chain-related A has been suggested. Although the first inflammatory process is unproven, cytotoxic activity by CD8+ T cells, γδ T cells, and natural killer cells, or the activation of dendritic cells (DC) and subsequent recruitment of immune cells by chemokines, seems to be the initial step. The recent discovery of anti-EPCR autoantibodies (Ab) and anti-SR-BI Ab suggests their roles in endothelial cell (EC) activation. Furthermore, anti-EC Ab (AECA) induces cytotoxicity, cytokine production, apoptosis, and mTOR pathway activation in EC. Subsequently, the recruited immune cells are activated. The activation of Th1 cells together with IL-12 and IL-18 secreted from DC releases tumor necrosis factor α and interferon γ, which activate macrophages, giant cells, and EC. The differentiation of Th17 cells is induced by IL-23, which is secreted by DC and macrophages. Anti-EPCR antibodies also promote Th17 cell differentiation, and Th17 cells promote neutrophil recruitment. Monocytes differentiate into macrophages and perform multiple effector functions. One of their functions is to promote inflammation by secreting chemokines, cytokines, reactive oxygen species (ROS), and reactive nitrogen intermediates (RNI). They also produce matrix metalloproteinase (MMP), which damages vascular components, and vascular endothelial growth factor (VEGF), which promotes neovascularization and subsequent inflammatory cell recruitment. Monocytes form granulomas together with neutrophils and T cells, and multinucleated giant cells are formed. Such inflammation disrupts the elastic lamellae, which contributes to artery dilatation. Giant cells and macrophages also produce growth factors, including platelet-derived growth factors and fibroblast growth factor-2 (FGF2), which trigger the migration of vascular smooth muscle cells into the intima, promote intimal proliferation of myofibroblasts, and produce matrix proteins, both of which contribute to artery stenosis. Tertiary lymphoid organs (TLOs) are formed in the adventitia, consisting of B cells, follicular dendritic cells (FDC), and high endothelial venules (HEV). Follicular helper T cells (Tfh) promote the maturation of B cells, and inflammation results in fibrous thickening and scarring of the adventitia. Ab, autoantibody; AECA, anti-endothelial cell antibody; DC, dendritic cell; EC, endothelial cell; EPCR, endothelial protein C receptor; FDC, follicular dendritic cells; HEV, high endothelial venule; IL, interleukin; IFN-γ, interferon γ; MMP, matrix metalloproteinase; mTOR, mammalian target of rapamycin; RNI, reactive nitrogen intermediates; ROS, reactive oxygen species; SR-BI, scavenger receptor class B type 1; T, T cells; Tfh, follicular helper T cells; TLO, tertiary lymphoid organ; TNFα, tumor necrosis factor α; VEGF, vascular endothelial growth factor.

The initial inflammation of TAK begins around the vasa vasorum, which leads to the infiltration of inflammatory cells around the border of the media and adventitia (Figure 1) ^(8), (9)^. Although the precise mechanism is unknown, the involvement of heat-shock protein 65 and major histocompatibility complex (MHC) class I chain-related A is suggested ^(10)^. Infiltrating cells of aortic tissue samples from TAK consist of macrophages, CD4+ T cells, CD8+ T cells, γδ T cells, and natural killer cells, and cell-mediated autoimmunity has been implicated in the pathogenesis of TAK ^(11)^. Although Ishikawa et al. hypothesized that TAK lesions begin in the left subclavian artery and subsequently extend to other sites ^(12)^, cluster analysis revealed that TAK lesions mostly develop symmetrically rather than contiguously in paired vascular beds ^(13)^. Therefore, failure of tolerance against the tissue around the vasa vasorum would be a critical pathogenesis in TAK, and the involvement of dendritic cells is also considered. Compared with giant cell arteritis (GCA), more CD8+ T cells, more CD20+ B cells, and a lower CD4/CD8 ratio were observed in TAK ^(14)^. Although granulomatous vasculitis is a typical pathological finding, T cells, including Th1 and Th17 cells, have been implicated as key players in systemic autoimmune responses ^(15), (16)^, and cytotoxic cells, including γδ T cells, natural killer cells, and CD8 T cells, secrete massive amounts of perforin ^(11)^. Myeloid cells, including macrophages, are effector cells that promote disease progression *via* several pathways (Figure 1) ^(15), (16)^. Intensive inflammation disrupts the elastic lamellae, where the presence of multinucleated giant cells and vasculitis is sometimes observed, thereby leading to dilatation of the affected artery. Intimal hyperplasia is also a major pathogenic mechanism in vasculitis, which occludes the vascular lumen and obstructs blood flow to dependent organs. The growth, migration, and secretory activity of smooth muscle cells that form the hyperplastic intima depend on appropriate growth factors ^(16)^. Inflammation results in fibrous thickening and scarring of the adventitia, which is more prominent in TAK than in GCA ^(17)^.

## 3. B Cells in TAK

Although the role of B cells in TAK has been controversial ^(13)^, recent evidence supports the involvement of B cells and humoral immunity in the pathogenesis of TAK (Figure 1). Particularly, greater infiltration of B cells was observed in TAK than in GCA ^(14)^. Fernandez et al. found enrichment in active chromatin epigenetic marks in TAK, with monocytes and B cells exhibiting the highest enrichment patterns ^(18)^. Clement et al. investigated tertiary lymphoid organs (TLOs) in the aortic wall of patients with TAK. B-cell aggregate-TLOs containing high endothelial venules (HEVs) were observed in the adventitia, and ectopic follicles containing CD21+ follicular dendritic cells were detected in active samples. Flow cytometry analysis confirmed the accumulation of memory/germinal center-like B cells in the adventitial layer and revealed the presence of antigen-experienced T follicular helper (Tfh) cells. Ectopic lymphoid neogenesis, displaying functional features, can be found in the aortic wall of a subset of patients with active TAK ^(19)^. Desbois et al. conducted transcriptome and phenotype analyses in peripheral blood and arterial lesions from patients with TAK; they found an increase in Tfh cells in both circulating and aorta-infiltrating CD4+ T cells, which promote B-cell maturation ^(20)^. An increase in Tfh cells was also found by Matsumoto et al. who reported that Tfh cells, along with other helper T cells, were associated with disease activity in TAK ^(21)^. Although the change in B-cell subsets was not significant in their study, Hoyer et al. reported that CD19+CD20-CD27 high antibody-secreting cells were significantly higher in active patients with TAK ^(22)^. Furthermore, the successful response to rituximab also supports the fundamental role of B cells in TAK ^(22), (23)^. B-cell activation was examined using serum samples. Zanwar et al. measured the levels of B-cell survival factor activation factor (BAFF) and a proliferation-inducing ligand (APRIL) in the sera of 50 patients; they found that the APRIL levels were elevated in TAK patients, especially in active disease ^(24)^. In their study, the BAFF levels were not elevated, which was inconsistent with a previous study in which Nishino et al. reported BAFF elevation in active TAK patients ^(25)^.

## 4. Autoantibodies in TAK

The finding of Abs in TAK dates back to the 1960s, and the presence of anti-aortic antibodies was documented using complement fixation and hemagglutination tests with homogenized human aorta ^(26)^. Anti-aortic antibodies were evaluated using different approaches, including enzyme-linked immunosorbent assay (ELISA) and immunoblotting, and their titers were significantly higher in TAK patients than in healthy controls ^(27)^. Collagenase treatment of the aorta resulted in a decline in anti-aortic antibody fixation in TAK patients, suggesting the role of collagen in the auto-antigenicity of aortic tissue ^(28)^. Although some articles did not demonstrate the presence of anti-aorta specific antibodies ^(29)^, Dhingra et al. demonstrated that 80% of sera from TAK patients immunoprecipitated a protein of 45 kDa ^(27)^. Antiphospholipid antibodies have also been reported in TAK ^(30)^. Since the 1990s, anti-endothelial cell antibodies (AECAs) have been recognized to be present in high numbers in TAK patients ^(31), (32), (33)^. Anti-annexin V antibody was shown to be positive in 36% of patients, 54% of whom possessed AECAs. The pathogenic effects of AECAs in TAK have been investigated, including endothelial cell activation ^(34)^, cytotoxicity ^(35)^, cytokine production, apoptosis ^(36)^, and activation of the mammalian target of the rapamycin pathway (Figure 1) ^(37)^. However, identification of the target antigens of AECAs in TAK has been difficult as the target antigens are located on the plasma membrane, making the extraction of proteins difficult in proteomics analysis. To overcome the weakness of proteomic analysis, we developed a novel expression cloning system to identify cell-surface autoantigens, a serological identification system for autoantigens using a retroviral vector and flow cytometry (SARF) ^(38), (39)^. The usefulness of SARF has been demonstrated in several diseases ^(38), (39), (40)^, and later, two distinct autoantigens were identified as targets in TAK, EPCR, and scavenger receptor class B type 1 (SR-BI) ^(7)^.

## 5. Anti-EPCR Ab and Anti-SR-BI Ab in TAK

The positivity and clinical characteristics of anti-EPCR Ab or SR-BI Ab were investigated in 52 TAK patients ^(7)^. The positivity rates for anti-EPCR and anti-SR-BI Abs were 34.6% and 36.5%, respectively, and most of them were single positive for either of the Abs. Furthermore, these Abs were measured in other autoimmune rheumatic diseases, and the sensitivity and specificity of two Abs for TAK among autoimmune rheumatic diseases were 67.3% and 98.0%, respectively. Essentially, distinct clinical characteristics were observed in patients with these Abs. Patients with anti-EPCR Abs tended to experience more strokes and had significantly higher frequencies of UC. Also, in these patients, fewer arteries were affected, and type II artery lesions were dominant. Patients with anti-SR-BI Abs were relatively older, and aortic regurgitation was infrequent. Contrarily, they exhibited high levels of inflammatory markers and a wider distribution of artery lesions. Patients without these Abs tended to require surgery, including aortic valve replacement.

The pathogenic potential of the identified Abs was also investigated ^(7)^. Both EPCR and SR-BI are expressed on the endothelium in the vasa vasorum, as well as in the intima of the aorta. The major ligands of each autoantigen are activated protein C and high-density lipoprotein for EPCR and SR-BI, respectively. Interestingly, both EPCR and SR-BI suppress endothelial activation after an inflammatory stimulus. Abs against EPCR and SR-BI block the function of the corresponding ligands, thus inhibiting the resolution of endothelial activation, which has the potential to maintain vascular inflammation (Figure 1). Furthermore, EPCR suppresses the differentiation of Th17 cells, and the expression of EPCR was induced in naïve T cells under Th17 differentiation conditions. Anti-EPCR Abs suppress these activities, thereby promoting Th17 differentiation. These results indicate that Abs identified in TAK contribute to its pathophysiology in many ways. Furthermore, serial measurement of these Abs suggested their potential use for monitoring disease activity in TAK ^(7)^. In TAK, the relapse is frequent, accounting for 60% of cases ^(41)^. However, the recent use of biologics helps reduce the dose of corticosteroids ^(42)^, tocilizumab (TCZ), which inhibits the interleukin 6 pathway and masks the levels of C-reactive protein, making it difficult to monitor the disease activity of TAK. Therefore, a new tool for monitoring disease activity is required.

## 6. Anti-EPCR Ab in UC

Because the condition of many patients (37.5%) with anti-EPCR Abs was complicated by UC, anti-EPCR Abs were measured in primary UC without TAK ^(7)^. Among 35 patients with primary UC, 68.6% were proven to be positive for anti-EPCR Abs ^(7), (43)^. Based on these results, international validation of anti-EPCR Abs in inflammatory bowel disease (IBD) was performed among 203 patients with IBD and 100 non-IBD controls recruited from Japan and the United States ^(44)^. A cell-based assay using EPCR-overexpressing cells was conducted to analyze the anti-EPCR Ab activity. The positive rate of anti-EPCR Ab was 24.5% in Crohn’s disease (CD), 77.2% in UC, and 0% in control participants when the cut-off value was set to mean + 3 standard deviation of titers among healthy controls. The anti-EPCR Ab titer tended to be higher in patients with left-sided and total colitis than in those with proctitis. Interestingly, the anti-EPCR activity was significantly higher in colonic CD and negative in ileal CD, indicating an association between colonic inflammation and anti-EPCR Abs. Furthermore, the anti-EPCR activities were significantly higher in younger patients with UC and correlated with endoscopic activities according to the Mayo endoscopic subscore. These results confirm the importance of anti-EPCR Abs in colonic inflammation and large vessels. Although the precise pathomechanism of anti-EPCR Abs in UC is still under investigation, several articles have proposed the involvement of EPCR in IBD ^(45), (46)^. In addition to vascular endothelial cells in the submucosal layer, other cells, including colonic epithelial cells, have been reported to express EPCR ^(47)^. The epithelial expression of EPCR is decreased in IBD, leading to the disturbance of tight junctions. Although EPCR plays a role in inhibiting cell adhesion molecules, chemokine production, and leukocyte adhesion, its expression is reduced in IBD, which promotes intestinal inflammation ^(46)^. Considering the essential roles of EPCR in intestinal inflammation, the pathogenic potential of anti-EPCR Abs in IBD needs to be elucidated.

## 7. Autoantibodies and B Cells in UC

**Figure 2.**
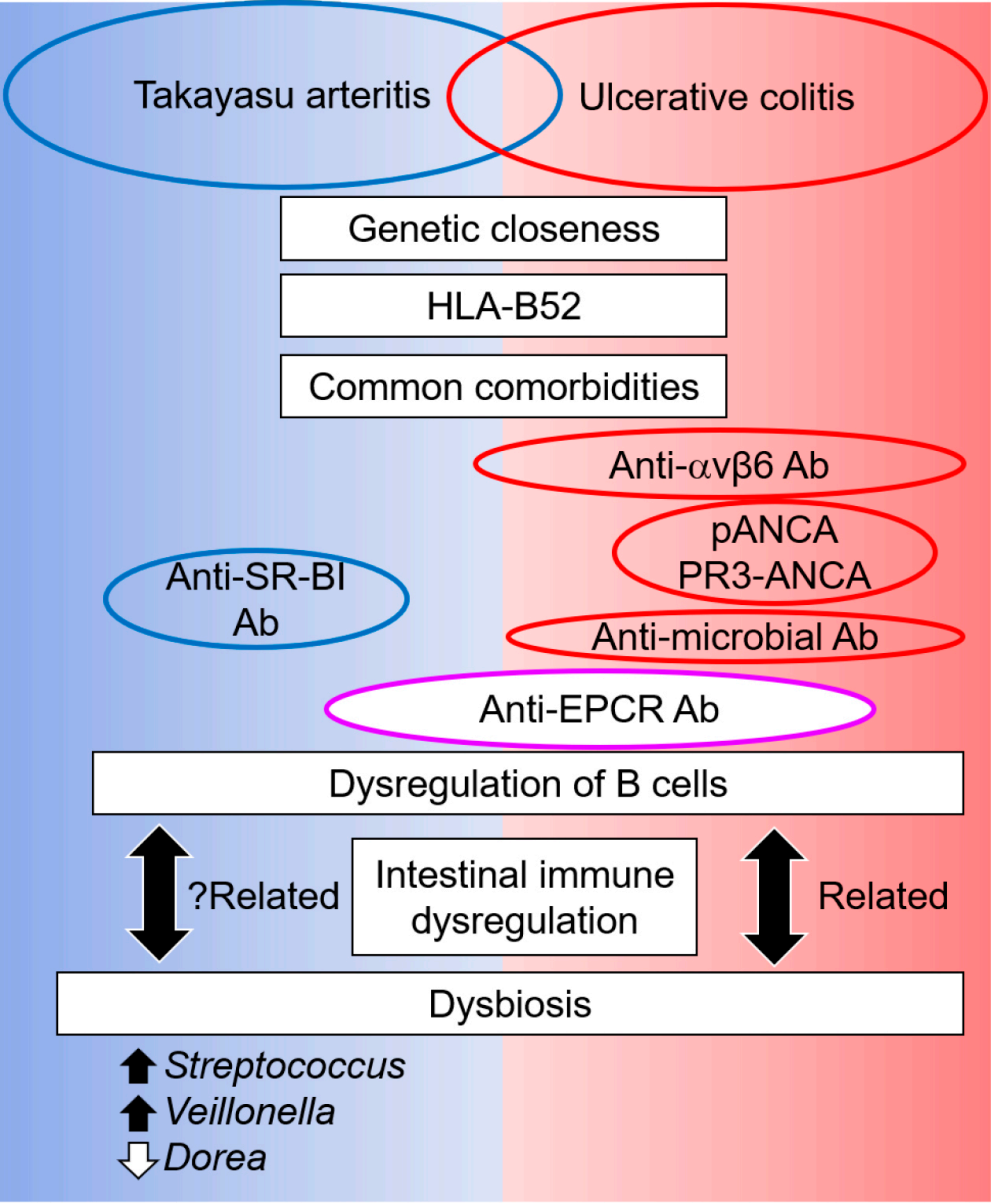
Comparison between Takayasu arteritis and ulcerative colitis: a possible role of gut-vessel connection in vascular inflammation Takayasu arteritis and ulcerative colitis sometimes coexist and share genetic risks and comorbidities. Although different antibodies appear, anti-EPCR autoantibodies are present in both diseases, and B-cell dysregulation is increasingly known. Dysbiosis is a critical pathogenesis in ulcerative colitis and is related to the dysregulation of intestinal immunity and, subsequently, B cells. Dysbiosis is also present in Takayasu arteritis, and such common features of ulcerative colitis suggest a relationship between dysbiosis and immune dysregulation in Takayasu arteritis. Ab, antibody; ANCA, anti-neutrophil cytoplasmic antibody; EPCR, endothelial protein C receptor; HLA, human leukocyte antigen; PR3, proteinase 3.

Identification of anti-EPCR Abs in UC supported the potential contribution of B cells to its pathogenicity, although the depletion of B lymphocytes by rituximab had no significant effect on the induction of remission in moderately active UC ^(48)^. As an antibody response in UC, anti-*Saccharomyces cerevisiae* antibodies and perinuclear anti-neutrophil cytoplasmic antibodies (ANCA) were investigated (Figure 2) ^(49)^. In addition, proteinase-3 ANCA, which is mostly specific for granulomatosis with polyangiitis, but sometimes becomes positive in other diseases ^(50), (51), (52), (53), (54)^, can be weakly positive in UC ^(55)^. The induction of anti-commensal immunoglobulin G (IgG) and activation of Fc gamma receptor signaling were also observed in UC ^(56)^. Subsequently, Kuwada et al. found novel Abs against integrin αvβ6 in UC by screening for autoantigens among integrin proteins using ELISA ^(57)^. The addition of Mg^2+^ and Ca^2+^, which are required for the heterodimer formation of integrin, significantly increased the positivity for anti-integrin αvβ6 activity between without and with Mg2+ and Ca2+ (73.4% and 95.3%, respectively). The positivity of anti-integrin αvβ6 Abs in UC differed according to the degree of mucosal damage, suggesting that Ab generation is a secondary event following epithelial cell destruction in UC. The common feature of anti-integrin αvβ6 and anti-EPCR Abs is that target antigens are expressed on the extracellular domain of the plasma membrane; therefore, they can manifest pathogenic functions, including direct cytotoxicity and interference of their ligands ^(43)^. In a mechanistic study, the serum IgG of patients with UC blocked integrin αvβ6-fibronectin binding through an RGD tripeptide motif and inhibited cell adhesion, which could affect mucosal healing ^(57)^.

As B cells have been understudied in UC, Uzzan et al. investigated the landscape of B cells during UC-associated intestinal inflammation ^(58)^. A previous study reported an increase in IgG1+ plasma cells (PCs) in the colonic tissue of UC ^(59)^. Furthermore, IgA+ PCs were the largest population within the gut PC compartment in UC, and a significant increase was observed in IgG+ PCs in the inflamed gut lamina propria of patients with UC. The number of cycling plasmablasts and recently recruited PCs has also increased. Interestingly, colonic IgG+ PCs and circulating plasmablasts in UC exhibited a significant reduction in VDJ gene mutations, which might be due to chronic antigen-mediated overstimulation of gut follicular B cells. The pathogenic B-cell response is associated with a subset of intestinal CXCL13-expressing Tfh-like T peripheral helper cells. Furthermore, the expansion of gut-homing plasmablasts was correlated with disease activity and reflected changes in humoral immunity in the intestine. Altogether, the B-cell response also seems to be highly dysregulated in UC.

## 8. Common Etiology among TAK and UC: A Possible Role of Gut-Vessel Connection in Vascular Inflammation

Although the target organs in TAK and UC are different, a specific population of patients share the same Abs against EPCR (Figure 2). This evidence suggests the presence of a common etiology among these diseases, which is associated with aberrant B-cell activation. The close clinical relationship between these diseases is supported by a recent genetic study, which demonstrated that the closest genetic relatedness of TAK was IBD, including UC, CD, and spondyloarthropathy, rather than other vasculitidis ^(18)^. In addition, TAK and UC are associated with human leukocyte antigen (HLA)-B52 ^(5)^. Among HLA-class I-related diseases, spondyloarthropathy is representative of rheumatologic diseases and is sometimes complicated by TAK and UC ^(60)^. Increasing evidence has suggested that the human gastrointestinal microbiome plays a critical role in the pathogenesis of IBD ^(61)^. Recent understanding regarding the dysregulation of B cells and production of specific Abs in UC has confirmed that dysbiosis interacts with such dysregulation of B cells, although it remains unclear which is the preceding event. In addition to IBD, gut dysbiosis and an altered immune response are associated and could contribute to the pathogenesis of some autoimmune diseases, including spondyloarthropathy ^(62)^. The similarity in the background and clinical characteristics of TAK with UC, and in part with spondyloarthropathy, suggests the involvement of dysbiosis in TAK.

However, information on the intestinal microbiota in TAK is limited. Desbois et al. evaluated the blood microbiome profile by sequencing 16S rDNA blood bacterial DNA ^(63)^. At a linear discriminant analysis threshold of >2, an increase in the levels of *Clostridia*, *Cytophagia*, and *Deltaproteobacteria* and a decrease in *Bacilli* at the class level were observed in TAK patients compared with healthy controls. However, these data were not comparable with those of two recent articles that investigated the fecal microbiome and showed common dysbiosis ^(64), (65)^. Fan et al. investigated dysbiosis and its crosstalk with phenotypes in TAK using shotgun sequencing of the fecal metagenome ^(64)^. Consistently altered microbial taxa included five genera: enriched *Streptococcus*, *Veillonella*, *Klebsiella*, and *Escherichia* and depleted *Dorea*. We also investigated the intestinal microbiota using 16S rRNA amplicon sequencing; the altered genera microbial taxa in our study included enriched *Streptococcus*, *Lactobacillus*, *Veillonella*, and *Enterococcus* and depleted *Bacteroides*, *Phascolarctobacterium*, *Dorea*, and *Parasutterella* ^(65)^. Therefore, the enrichment of *Streptococcus*, *Veillonella*, and depleted *Dorea* was a common dysbiosis observed among patients with TAK in different social environments and might be specific microbial features ^(66)^, although further validation is required (Figure 2). Nonetheless, commonly altered intestinal microbiota is relevant in the exacerbation of inflammation, and dysbiotic microbes are also directly or indirectly linked to TAK phenotypes *via* metabolic and lipid modules ^(64)^. Such alterations in the intestinal microbiota have the potential to cause complications in TAK. In particular, the prevalence of oral microbes increased and became a causative organism in patients with TAK complicated by infectious endocarditis during TCZ treatment ^(67)^. Interleukin 6 plays physiological roles in the intestine, and blocking its roles sometimes causes undesired adverse events in UC ^(68)^ and some susceptible TAK patients ^(69)^.

In spondyloarthritis, in which dysbiosis is associated with arthritis, the gut-joint axis through the generation of an arthritogenic peptide and expression of homing molecules has been proposed ^(62)^. Although the mechanisms by which dysbiosis contributes to vascular inflammation need to be investigated, similar pathways are possible candidates, and the mechanism of anti-EPCR Abs generation might be important. In addition to previous knowledge regarding the roles of T cells and myeloid cells in TAK, investigation of the interaction between dysbiosis and dysregulation of B cells and Abs is required for a comprehensive understanding of the complex pathophysiology of TAK.

## Article Information

The article is based on the study, which received the Medical Research Encouragement Prize of The Japan Medical Association in 2022.

### Conflicts of Interest

None

### Sources of Funding

This work was partly supported by JSPS KAKENHI Grant Number 21K08469T and the JCR Grant for Promoting Research for D2T RA.

### Author Contributions

TS designed and drafted the manuscript.
